# Orbital Presentation of Eosinophilic Granulomatosis with Polyangiitis: An Interventional Case Report and Literature Review

**DOI:** 10.18502/jovr.v20.16399

**Published:** 2025-08-27

**Authors:** Ozlem Barut Selver, Emil Ahmadli, Muhammed Dara Tas, Banu Yaman, Naim Ceylan, Mozhgan Rezaei Kanavi

**Affiliations:** ^1^Department of Ophthalmology, Ege University Faculty of Medicine, Izmir, Turkey; ^2^Ocular Surface Research Laboratory, Ege University, Faculty of Medicine, Izmir, Turkey; ^3^LimbuStem R&D Medical Products, Izmir, Turkey; ^4^Institute of Health Sciences, Department of Stem Cell, Ege University, Izmir, Turkey; ^5^Cord Blood Cell-Tissue Application and Research Center, Ege University, Izmir, Turkey; ^6^Department of Pathology, Ege University Faculty of Medicine, Izmir, Turkey; ^7^Department of Radiology, Ege University Faculty of Medicine, Izmir, Turkey; ^8^Ocular Tissue Engineering Research Center, Research Institute for Ophthalmology and Vision Science, Shahid Beheshti University of Medical Sciences, Tehran, Iran

**Keywords:** Antineutrophil Cytoplasmic Antibody, Eosinophilia, Eosinophilic Granulomatosis with Polyangiitis, Orbit, Systemic Steroid

## Abstract

**Purpose:**

To report a case of eosinophilic granulomatosis with polyangiitis (EGPA) initially presenting as orbital involvement, describe its successful management, and provide a comprehensive literature review.

**Case Report:**

A 33-year-old female patient presented with swelling, redness, tenderness, and a mass under the left upper eyelid for one month. Upper lid eversion showed a multilobulated lesion in the subconjunctival area of the same region. The patient's medical history included asthma and atrial septal defect surgery. Orbital MRI revealed a soft tissue mass infiltrating the superior and lateral aspects of the conal and extraconal regions in the anterior orbit, with extension toward the preseptal area. The lesion underwent incisional biopsy, and histopathological findings were consistent with the diagnosis of EGPA. The patient's blood tests revealed eosinophilia and a negative antineutrophil cytoplasmic antibody. After excluding other similar pathologies such as granulomatosis with polyangiitis, we observed a dramatic regression in her orbital lesion following systemic steroid therapy.

**Conclusion:**

The diagnosis of EGPA, a rare clinical presentation, is crucial for ophthalmologists because it provides early recognition of the systemic disease and can help slow its progression by initiating appropriate treatment in a timely manner.

## INTRODUCTION

Eosinophilic granulomatosis with polyangiitis (EGPA), formerly known as Churg-Strauss syndrome, is a rare disease that typically begins with asthma and eosinophilia, progressing to necrotizing vasculitis in small- to moderate-sized vessels.^[1,2]^

The disease begins in a prodromal phase, characterized by poorly controlled asthma, sometimes accompanied by systemic allergy, and progresses to an eosinophilic phase marked by peripheral eosinophilia and eosinophilic infiltration of multiple organs. The last phase is the vasculitic phase, which occurs approximately 8 to 10 years after the prodromal phase and has a high mortality rate.^[3]^

In EGPA, the ocular manifestation is unusual and generally manifests as orbital inflammation or ischemic vasculitis. Orbital inflammation presents as dacryoadenitis, myositis, perineuritis, conjunctival granuloma, and episcleritis, which tend to progress to a more chronic state and lead to negative antineutrophil cytoplasmic antibody (ANCA) test results.^[4,5,6]^

Here, we report a case of EGPA that initially presented as orbital involvement, discuss its successful management, and conduct a detailed literature review. The purpose of reporting this case is not only to add to the current literature but also to highlight anterior orbital involvement as a rare presentation of EGPA. This report may be helpful for ophthalmologists to consider this pathology in the differential diagnosis of a patient presenting with an anterior orbital lesion. Our case report adheres to the tenets of the Declaration of Helsinki.

## CASE PRESENTATION

**Figure 1:**
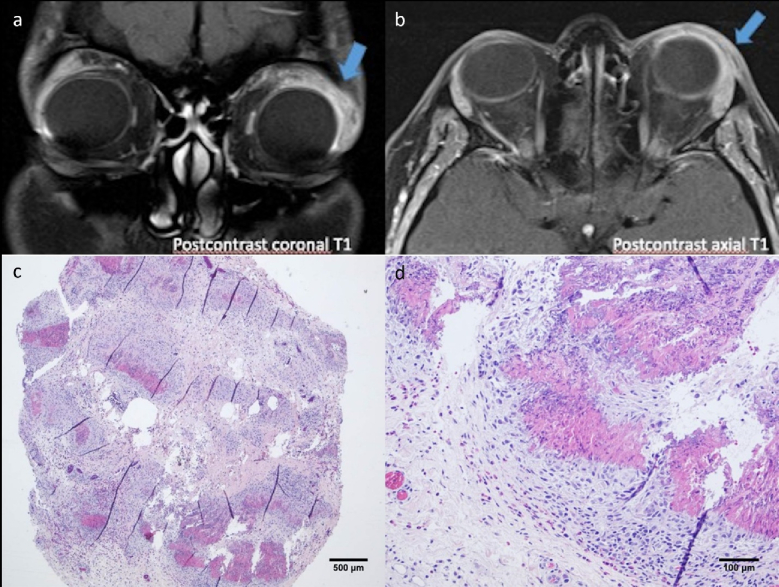
Ocular presentation of eosinophilic granulomatosis with polyangiitis in the reported patient. Note the presence of left upper eyelid swelling, redness, and mass (a). A multilobulated lesion is observed in the subconjunctival region of the superior fornix (b). Pre (c)- and post (d)-treatment images of the orbital eosinophilic granulomatosis with polyangiitis in the presented case. Note the significant regression of the infiltrative orbital lesion following oral steroid therapy.

A 33-year-old female patient was admitted with weekly swelling, redness, tenderness, and a mass under the left upper eyelid for one month [Figure 1a].

**Figure 2:**
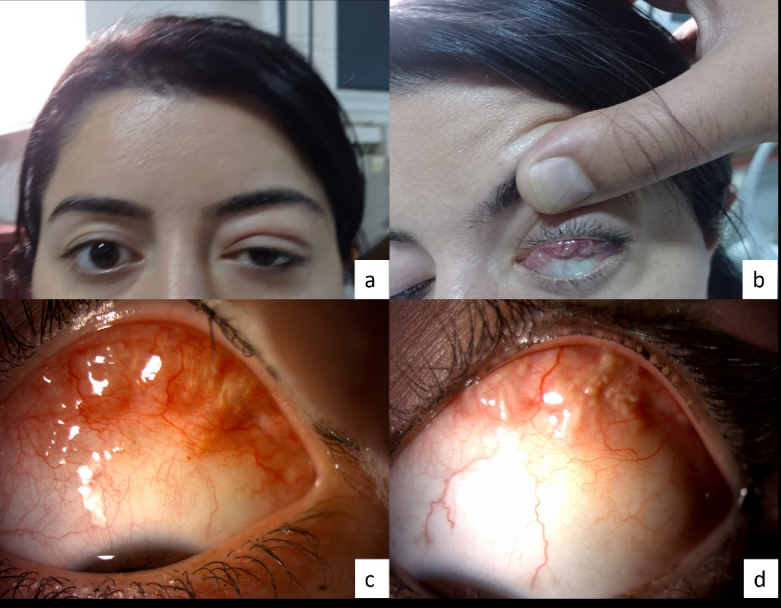
Orbital MRI and representative histopathological images of the orbital biopsy in the reported case. Note the presence of a soft tissue mass infiltrating the superior and lateral aspects of the conal and extraconal regions of the orbit, extending toward the preseptal area (arrows) in the post-contrast coronal (a) and axial (b) sections. Image (c) represents infiltration of fibro-fatty tissue by eosinophilic granulomatous areas. Image (d) illustrates a higher magnification of an eosinophilic granulomatous infiltration consisting of histiocytes and a few multinucleated giant cells surrounding an eosinophilic amorphous material. Note the presence of eosinophil-rich inflammatory cell infiltration around the granulomatous lesion (Hematoxylin and eosin stain).

Downward gaze disclosed a multilobulated lesion in the upper forniceal subconjunctival area [Figure 1b]. Apart from this, bilateral anterior and posterior segment images were normal. The patient had a seven-year history of asthma, chronic sinusitis, and cardiac surgery due to a congenital atrial septal defect. Orbital magnetic resonance imaging (MRI) revealed a soft tissue mass infiltrating the superior and lateral aspects of the conal and extraconal regions in the anterior orbit and extending toward the preseptal area [Figure 2a & 2b].

An incisional needle biopsy was obtained from lesions in the upper lid fornix for diagnostic evaluation. Histopathological examination revealed an eosinophilic granulomatous inflammation composed of histiocytes and multinucleated giant cells surrounding an eosinophilic amorphous material. It also indicated increased vascularity and eosinophil-rich inflammatory cell infiltrates around the granulomatous lesion. These findings were consistent with the diagnosis of EGPA [Figure 2c & 2d].

MRI imaging was compatible with bilateral ethmoid and maxillary sinusitis. Lung imaging revealed hilar enlargement, but no granuloma.

In the patient's blood tests, eosinophilia was present in the hemogram (19.9%), IgG4 levels were within the normal range (1.28 g/L), and the ANCA test result was negative.

After excluding other similar pathologies, such as Wegener's, oral prednisolone 1 mg/kg/day was initiated, and a dramatic response in lesion size was observed in an eight-week follow-up [Figure 1c & 1d]. Oral prednisolone was then tapered by 8 mg every two weeks and then maintained at 4 mg per day. The patient continued to take 4 mg oral prednisolone per day.

Given the patient's history of cardiac surgery, the attending physician overseeing her care was informed about the diagnosis to monitor her for future cardiac complications. Although the patient's ophthalmological examinations were conducted in our department, other investigations for systemic workup and organ involvement were performed at different centers. Therefore, we had limited access to non-ophthalmic findings, such as imaging performed for systemic evaluation. Written informed consent was obtained from the patient for the publication of images and corresponding clinical information.

## DISCUSSION

First described by Churg and Strauss in 1951, EGPA is a small to moderate-sized eosinophilic vasculitis.^[7]^ It is indeed classified as idiopathic, meaning its exact cause remains unclear. However, recent research has shed light on some of the molecular mechanisms and immunological processes involved in the disease, particularly the role of T-helper 2 (Th2) cells.^[4]^ It is known that certain cytokines, such as Th2-associated IL-4 and IL-13, are produced, and these cytokines promote the synthesis of an exotoxin that recruits eosinophils. Activated tissue eosinophils secrete significant amounts of eosinophil granule protein, which accumulates in the tissue and directly leads to organ damage and necrotizing granulomas.^[8]^ In addition, ANCAs are also an important component in the pathogenesis of the disease. It is suggested that these antibodies activate neutrophils, causing the release of cytokines and proteins that damage the tissue and endothelium.^[9]^

When examining the clinical presentation of the disease, it typically develops in three stages. The first phase is the prodromal asthma and allergic phase, which usually occurs in the second and third decades of life. The next (eosinophilic) phase includes peripheral eosinophilia and eosinophilic infiltration of many organs. This condition typically affects the lungs and gastrointestinal tract, manifesting as pulmonary opacities, asthma, or gastroenteritis. The last stage is characterized by life-threatening vasculitis, in which small- and medium-sized vessels are affected. This phase occurs approximately a decade after the asthma phase and is often associated with extravascular granulomas.^[4]^

Orbital involvement in EGPA is less common but may pose specific challenges in diagnosis and management. This case report describes a case of ANCA-negative EGPA initially presenting with orbital involvement. Treatment modalities may vary from topical corticosteroids in non-vision-threatening inflammations to high-dose corticosteroids and immunosuppressants in the severe forms of ocular involvement.^[11]^ Our patient with the orbital presentation of the disease demonstrated a dramatic response to oral steroid therapy.

In terms of epidemiology, the prevalence of EGPA is approximately 1 per 100,000.^[12]^ Ocular and orbital inflammation were reported in 11% and 0.7% of EGPA cases, respectively.^[10]^ The disease typically peaks at ages 30–40 or 55–64, with no gender or ethnic predominance.^[13]^ However, the reported mean age in ocular EGPA was 49.3 years, with a male-to-female ratio of 1.42.^[4]^ Our patient was a 33-year-old female, falling within the expected first peak range of EGPA.

Ocular EGPA develops in two primary clinical forms: ischemic vasculitis or idiopathic orbital inflammation. Ischemic vasculitis involvement is more likely to present in unilateral disease than the inflammatory type. It is also reported to affect the older population, tends to present with more aggressive disease requiring immunomodulators, and often has worse visual outcomes. Differences in age, prognosis, and treatment modalities are due to the fact that while the inflammatory type represents the early stage of EGPA with a more benign prognosis, the ischemic type represents the advanced stage of vasculitis.^[14]^ Common manifestations of orbital inflammation include conjunctival nodules (40%), orbital myositis (25%), and orbital inflammatory syndrome (20%). On the other hand, ischemic vasculitis may present with retinal artery and vein occlusions (48%), ischemic optic neuropathy (32%), and retinal vasculitis or edema (2.8%).^[15]^ The most common presenting symptoms of orbital involvement are redness (30%), periocular swelling (25%), diplopia (20%), foreign body sensation (5%), and eyelid lesions (5%).^[4]^ Our case presented with orbital inflammation, periocular swelling in the left eye, and a multilobular subconjunctival nodule extending posteriorly in the superior fornix.

According to the American College of Rheumatology, the diagnosis of EGPA requires the presence of four or more of the following six conditions: asthma, eosinophilia > 10%, neuropathy, migratory or transient pulmonary opacities, paranasal sinus abnormalities, and extravascular eosinophils on tissue biopsy. Our case met four of the diagnostic criteria for EGPA, including asthma, eosinophilia (19%), tissue eosinophilia, and paranasal sinus abnormality.^[16]^

In cases with suspected systemic EGPA, the ANCA test is recommended for diagnosis and follow-up.^[17]^ ANCA positivity ranges from 30% to 40% in patients with EGPA. A similar rate of 33% has been reported in ocular EGPA.^[7]^ Since ANCA positivity is associated with ischemic presentations, it is crucial to follow up patients for potential complications, including retinal artery and vein occlusions, ischemic optic neuropathy, and retinal vasculitis. Although our patient had a history of congenital heart disease not related to EGPA, attention should be paid to the possibility of cardiac involvement in ANCA-negative cases in the future.^[18]^ Given her history of cardiac surgery, we suggested that the supervising physician responsible for the patient's follow-up address this concern.

The differential diagnosis of ophthalmic EGPA includes hypereosinophilic syndrome, granulomatosis and polyangiitis, microscopic polyangiitis, and parasitic infections. Therefore, a team of ophthalmologists, internists, and/or rheumatologists is required to arrive at the correct diagnosis.^[4]^ In our patient, these differential diagnoses were carefully excluded.

To manage a patient with EGPA, it is vital to determine the severity of the disease based on a five-factor scoring (FFS) system that considers involvement of the central nervous system, gastrointestinal tract, heart, and kidneys—with renal involvement described as 24-hour proteinuria > 1 g and a serum creatinine > 140 μ mol/L. Each of these five criteria is given 1 point.^[19]^ In the absence of organ involvement, the FFS is 0, and the primary treatment is to administer systemic steroids. The regimen starts with oral prednisolone at doses of 0.5–1.5 mg/kg/day for 6 to 12 weeks or until remission is achieved, followed by a gradual taper. This is typically effective in cases with ophthalmic EGPA of the idiopathic orbital inflammatory type.^[4,19]^ After the diagnosis was confirmed and necessary information was given to our patient, oral systemic steroid treatment at a dose of 1 mg/kg was started, and a dramatic clinical improvement in the orbital lesion was observed at the follow-up.^[10]^ When the FFS is ≥ 1, the patient has severe organ involvement, and a combination of corticosteroids and immunosuppressives should be administered for at least 24 months after remission.^[19,20]^ Patients with EGPA who have an FFS > 1 can benefit from other treatment modalities such as plasma exchange, intravenous immunoglobulins, interferons, mepolizumab, and rituximab.^[21]^

Ophthalmic EGPA, although rare, is clinically important in that it can be initially diagnosed by ophthalmologists. In the literature, only 35.6% of identified patients with EGPA were referred to ophthalmologists; however, in the majority of cases, including our case, the diagnosis was first made by an ophthalmologist.^[4]^ A team approach involving ophthalmologists, internists, and/or rheumatologists is necessary for the accurate diagnosis and management of EGPA. Since untreated EGPA has a high risk of mortality in the vasculitic process, proper diagnosis and management are vital in reducing the mortality rate.

It is reported that half of EGPA-related death rates are due to cardiac disorders, and approximately one-fourth of patients with ophthalmic EGPA have cardiac involvement at the time of ocular presentation.^[4]^ Accordingly, these patients should receive not only ocular management but also cardiac evaluation, given its clinical importance. In our case, the history of cardiac disease dated back to childhood and was not related to EGPA. However, the question remains whether EGPA, despite proper treatment, might exacerbate her cardiac problem in the future.

The limitation of this report was that the patient was lost to follow-up when she was referred for investigation of involvement in other organs. Therefore, we were unable to provide exact information on other organ involvement or changes in the sinus and pulmonary areas after starting steroid therapy.

In summary, recognition of EGPA, a rare clinical presentation, by ophthalmologists enables early diagnosis of the disease and is critical in slowing its progression through the timely initiation of appropriate treatment.

## Financial Support and Sponsorship

None.

## Conflicts of Interest

None.
